# Snakebites notified to the poison control center of Morocco between 2009 and 2013

**DOI:** 10.1186/s40409-016-0065-8

**Published:** 2016-03-16

**Authors:** Fouad Chafiq, Faiçal El Hattimy, Naima Rhalem, Jean-Philippe Chippaux, Abdelmajid Soulaymani, Abdelrhani Mokhtari, Rachida Soulaymani-Bencheikh

**Affiliations:** Centre Anti Poison et de Pharmacovigilance du Maroc, Rabat, Maroc; Laboratoire de Génétique et Biométrie Faculté des Sciences, Université Ibn Tofail, Kénitra, Maroc; UMR 216, Mère et enfant face aux infections tropicales, Institut de Recherche pour le Développement, Cotonou, Bénin, and Université Paris Descartes, Sorbonne Paris Cité, Faculté de Pharmacie, Paris, France; Faculté de Médecine et de Pharmacie, Université Mohammed V, Rabat, Maroc

**Keywords:** Snakebite, Envenomation, Viper, Morocco, Epidemiology

## Abstract

**Background:**

Snakebites cause considerable death and injury throughout the globe, particularly in tropical regions, and pose an important yet neglected threat to public health. In 2008, the Centre Anti Poison et de Parmacovigilance du Maroc (CAPM) started to set up a specific strategy for the control of snakebites that was formalized in 2012. The aim of the present study is to describe and update the epidemiological characteristics of snakebites notified to CAPM between 2009 and 2013.

**Methods:**

This retrospective five-year study included all cases of snakebites notified to CAPM by mail or phone.

**Results:**

During the study period, 873 snakebite cases were reported to CAPM, an average incidence of 2.65 cases per 100,000 inhabitants with 218 cases each year. The highest incidence was found in Tangier-Tetouan region with 357 cases (40.9 %) followed by Souss Massa Draa region with 128 cases (14.6 %). The average age of patients was 26.8 ± 17.2 years. The male to female sex ratio was 1.67:1 and 77 % of cases occurred in rural areas. The bites occurred mainly in spring (44 %) followed by summer (42 %). Snake species was identified in 54 cases (6.2 %): colubrids represented 31 % (*n* = 18) and vipers 67 % (*n* = 36), mainly *Daboia mauritanica*, *Bitis arietans* and *Cerastes cerastes*. In 311 cases (35.6 %), the patients showed viper syndrome. Thrombocytopenia was observed in 23.5 % of viper syndrome cases, whereas, compartment syndrome was observed in 7.6 % patients. FAV-Afrique® was administered in 41 patients (5 %). In patients treated with antivenom, 38 patients recovered and three died. Twenty-seven deaths were reported (3.9 %).

**Conclusion:**

Despite specific efforts to better understand the epidemiology of snakebites in Morocco (incidence, severity, snake species involved), it remains underestimated. Therefore, further work is still necessary to ensure accessibility of appropriate antivenom against venomous species and to improve the management of envenomation in Morocco.

## Background

In Morocco, the incidence of snakebite envenomation is estimated at 0.34 per 100,000 inhabitants and case fatality rate reaches 7.2 % [1]. Although the number of victims is elevated, it remains a neglected disease, since it is under reported when compared to scorpion stings, whose incidence is much higher. Usually, two snake families are involved in accidents: seven species from the Viperidae family are found throughout the country (*Daboia mauritanica*, *Bitis arietans*, *Cerastes cerastes*, *Cerastes vipera*, *Echis leucogaster*, *Vipera latastei* and *Vipera monticola*), while the family Elapidae is represented by a single species, *Naja haje legionis* [2–4].

Regarding the type of envenomation, vipers are responsible for the viper syndrome that associates inflammatory and hemorrhagic disorders. Elapids are responsible for a neurotoxic syndrome characterized by respiratory paralysis [5]. Since 2008, the Centre Anti Poison et de Parmacovigilance du Maroc (CAPM) has been developing a specific strategy for controlling snakebites. The main tasks are: implement a specific snakebite information system for data collection, standardize guidelines of snakebite treatment, acquire the antivenom FAV-Afrique®, which was not available prior to 2012, train medical and paramedical personal, and train physician toxicologists of CAPM to identify snakes and sensitize and inform the population. The aim of this study was to describe and update the epidemiological characteristics of snakebite envenomation reported to CAPM between 2009 and 2013.

## Methods

This is a descriptive and retrospective study over a period of five years (2009-2013), involving all snakebite cases reported to CAPM. Cases were recorded by three different forms and reporting methods established in August 2012: Intoxication Declaration Form (IDF), Information Toxicological Form (ITF) and a copy of the specific hospital report. Only the form ITF is filled based on phone calls from both public and health professionals.

The statistical analysis included the frequency of bites, age according to the International Programme on Chemical Safety classification [6], gender, origin of the patient (rural or urban region), season, clinical gradation, identification of the snake species if available, and clinical evolution. Patients with viper syndrome were graded according to the following severity criteria [7]:

- Grade 0 or dry bite: moderate pain, fang marks, without edema.
- Grade 1: severe pain, swelling not exceeding the elbow or knee.
- Grade 2: edema exceeding the elbow or knee.
- Grade 3: edema reaching or exceeding the root of the limb.

Snakebites were considered asymptomatic in cases without pain and fang mark. Decrease in the number of blood platelets (thrombocytopenia) was investigated in case of viper syndrome. Data on coagulation tests (WBCT, fibrinogen) are not requested routinely and very rarely investigated. We also analyzed the cases that received immunotherapy: FAV-Afrique® (produced by Sanofi Pasteur, France), the only antivenom available in Morocco since August 2012. It is a polyvalent antivenom composed of highly purified fragments of F(ab’)^2^ immunoglobulins against ten snake species among the most dangerous in Africa and belonging to families Viperidae and Elapidae. The initial dose recommended is 1 to 2 vials diluted to a total of 250 milliliters in isotonic fluid and infused over 30 to 60 min. The identification of snakes was carried out by photos taken by physicians or relatives of the victims who sent the images by e-mail to CAPM or even by evaluation of the dead animal. The initial characterization was performed by a trained toxicologist physician of CAPM specialized in ophidian identification and, then, confirmed by researchers of Rabat Scientific Institute (Morocco). Species formally recognized by a physician were also recorded.

## Results

**Fig. 1:**
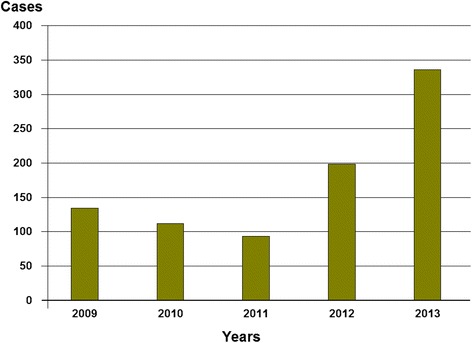
Number of snakebites according to years, from 2009 to 2013 (data from CAPM)

During the study period, 873 cases of snakebites (annual mean of 218 cases) were notified to CAPM, showing an average annual incidence by 2.65 cases per 100,000 inhabitants. The highest number of cases was registered in 2013 with 336 cases (40 %) (Fig. 1). The annual variation according to the regions of Morocco is specified in Table 1. The highest annual incidence was observed in the far north of the country in the region of Tangier-Tetouan (13.03 cases per 100,000 population) and that of Meknes-Tafilalet (5.46 cases per 100,000 inhabitants). In South, Souss Massa is also a risk area and has recorded 128 cases (3.74 cases per 100,000 inhabitants).

**Table 1:** Annual incidence and mortality according to the Moroccan regions (data from CAPM, 2009–2013)

	Number of bites	Population (×1,000)	No. of deaths	Incidence (per 100,000)	Mortality (per 100,000)	Lethality (%)
Chaouia-Ouardigha	15	1721	1	0.87	0.06	6.67
Doukala-Abda	2	2064	0	0.10	0.00	0.00
Fès-Boulemane	30	1723	4	1.74	0.23	13.33
Gharb-Chrarda-Beni Hssen	2	1982	0	0.10	0.00	0.00
Grand Casablanca	13	3855	1	0.34	0.03	7.69
Guelmim-Es Semara	20	522	1	3.83	0.19	5.00
Laayoune-Boujdour-Sakia El hamra	4	327	0	1.22	0.00	0.00
Marrakech-Tensift-Al Haouz	38	3304	5	1.15	0.15	13.16
Meknes-Tafilalt	123	2252	3	5.46	0.13	2.44
Oued Ed-Dahab-Lagouira	3	259	0	1.16	0.00	0,00
Rabat-Salé-Zemmour-Zaer	39	2695	2	1.45	0.07	5.13
Oriental	63	2003	1	3.15	0.05	1.59
Souss-Massa-Daraa	128	3421	6	3.74	0.18	4.69
Tadla-Azilal	28	1497	3	1.87	0.20	10.71
Tanger-Tetouan	357	2740	0	13.03	0.00	0.00
Taza-Al Hoceima-Taounate	5	1858	0	0.27	0.00	0.00

**Fig. 2:**
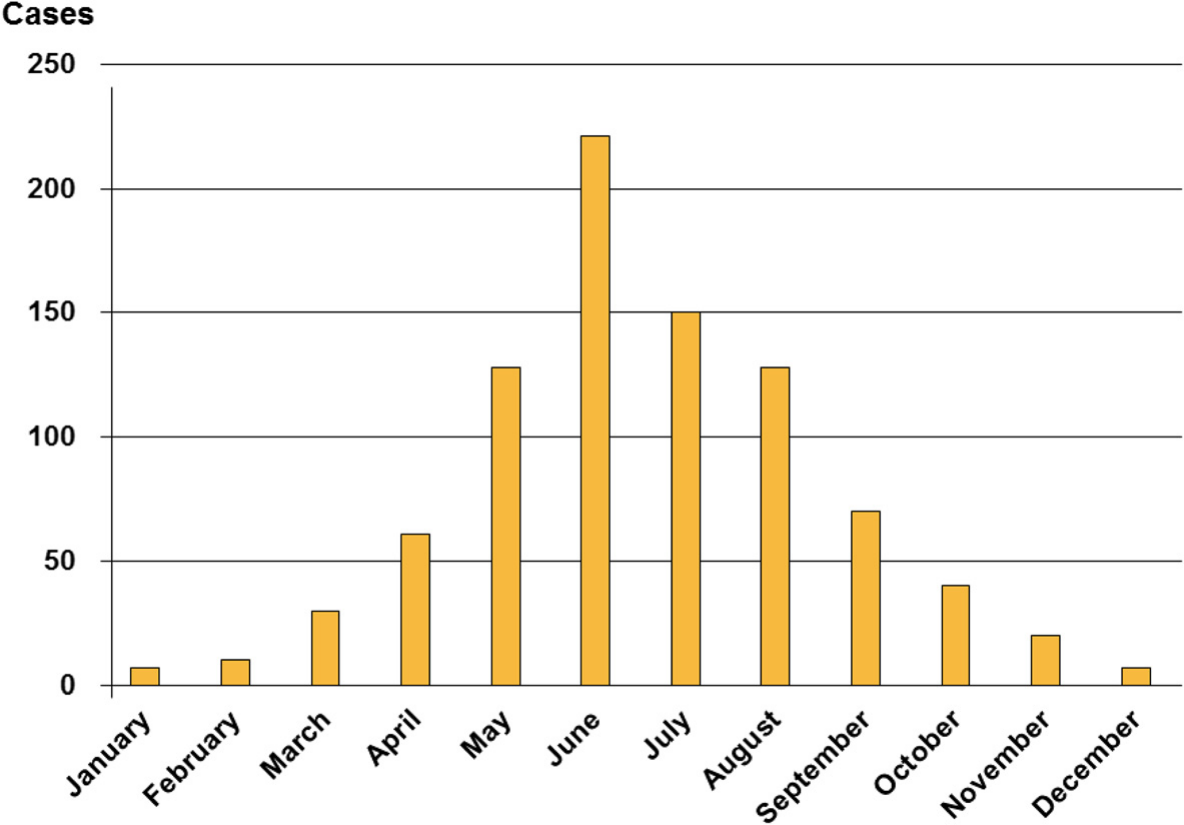
Monthly distribution of snakebites (data from CAPM, 2009-2013)

The mean age of the patients was 26.8 ± 17.2 years. Adults (>15 years old) represented 55.6 % of the cases. The male-female sex ratio was 1.67:1, and 77 % of cases occurred in rural areas. The bites occurred mainly in spring (44 %) and summer (42 %). June represented the peak of bites (25.3 %) (Fig. 2).

Snakes were identified in 54 cases (6.2 %). In 20.37 % of them, the dead snake was taken to CAPM for identification. The photo of the offending snake was sent via e-mail to CAPM in 72.22 % of the cases whereas the snake was formally recognized by a physician in 7.40 %. Colubrid snakes were involved in 33.4 % of the bites whereas vipers attacked 66.6 % of the victims, namely the species *Daboia mauritanica* (69.4 %), *Bitis arietans* (16.6 %) and *Cerastes* spp. (16.6 %). Out of 873 cases of snakebites, symptoms were specified in 502 cases (57.5 %) from which 311 cases were the viper syndrome. Sixty asymptomatic snakebites (6.8 %) were reported, including 18 after colubrid snakebites. Mild or severe envenomations involved 16.4 % of Grade 0 or dry bite, 33.4 % of Grade 1, 25.4 % of Grade 2 and 24.8 % of Grade 3. Thrombocytopenia was observed in 23.5 % (*n* = 165) and compartment syndrome in 7.6 % of cases. The overall case fatality rate was 3.9 % (27 deaths). Souss-Massa Draa recorded the highest number of deaths (six cases). A higher case fatality rate occurred in children aged between 5 and 14 years (12 deaths), showing a significant death risk [RR = 2.58; 95 % CI (1.18–5.62)].

FAV-Afrique® antivenom was administered to 41 patients (4.7 %) in doses ranging from 1 to 5 vials by patients. The average dose administered was 1.95 vials per patient. Among the patients treated with antivenom, the outcome was good in 38 patients while three died. One mild immediate adverse reaction (2.4 %) has been reported (tachycardia). One of the cases reported to CAPM was a 3-year-old male toddler presented to the emergency department three hours after a snake had bitten his lower right leg. He was unconscious and agitated. His family took the dead snake to CAPM that identified it as *Daboia mauritanica*. He was admitted to ICU presenting Grade 3 viper syndrome, with thrombocytopenia and anemia. He was intubated and ventilated. The brain scan revealed a subarachnoid hemorrhage. The antivenom was not available locally, only a hospital 250 km away could offer two vials of FAV-Afrique®. One vial was given 30 h after the snakebite and another one five days after without clinical or biological improvement. The toddler died ten days after the snakebite.

In our study, the analysis of the clinical evolution did not show a significant association (*p* > 0.05) between the death and the administration of FAV-Afrique® (Table 2).

**Table 2:** Outcome according to the administration or not of the antivenom FAV-Afrique® (data from CAPM, 2009-2013)

	Favorable	Death	*χ* ^2^	*p*	Relative risk	Confidence interval (95 %)
FAV Afrique®						
Yes	38	3	3.34	>0.05	0.3	0.079- 1.151
No	46	12				
Total	84^a^	15^a^				

^a^Total cases where there is information about outcome and administration of FAV-Afrique®

## Discussion

In Africa, mostly in sub-Saharan Africa, snakebite comprises a neglected public health problem that affects one million people every year, resulting in 100,000 to 500,000 envenomations and 10,000 to 30,000 deaths [8]. These envenomations have an impact on morbidity due to the abundance of snakes that affect agricultural activities, poor availability and accessibility of antivenoms [9]. In Morocco, our study showed that the average is more than 200 snakebites per year whereas the case fatality rate is nearly 4 %. Although far behind the scorpion stings, envenomation by snakebites remains underestimated and a neglected public health issue [10]. A long-term study, between 1992 and 2007, showed of a diminished number of notifications (100 cases per year) [11].

The creation of a control strategy in 2008 did not significantly change the pattern of notification until 2011. However, since 2012, the number of registered cases increased expressively (about 50 % in 2012 and up to 150 % in 2013, compared to the years before 2012). An increase in the number of snakebites is unlikely, especially in consecutive years. However, the formalization of a strategy for snakebite management and awareness of both health staff and general population, which was conducted since 2012, certainly enhanced the case notification system. It is possible that in the future more cases will be reported.

Snakebites were predominant in rural areas, which suggests that the risk of bites may be higher because of more intense agricultural activities nowadays. This observation corroborates a previous study conducted by Chippaux in sub-Saharan Africa [8]. Mainly due to their agricultural activities, men were more afflicted with bites than females. Similar results have been reported by many studies in Africa and Asia [8, 9, 11, 12]. The snakebite increased in the spring and summer, resulting from higher activity of both snakes and humans [12, 13]. The highest incidence in Morocco’s northern regions can be explained by better reporting of cases due to better management of the health system. The other risk areas are Meknès-Tafilat in the northeast and Souss Massa Draa in the south of Morocco. This high incidence could be explained by their large population, but also by the diverse fauna of snakes found there. Furthermore, the climate in south area that varies from semiarid to arid may play an important role in this distribution of snakes and, therefore, snakebites.

Two families of venomous snakes are found in Morocco: Viperidae that consists of seven species found throughout the country – *Daboia mauritanica*, *Bitis arietans*, *Cerastes cerastes*, *Cerastes vipera*, *Echis leucogaster*, *Vipera latastei*, *Vipera monticola*; and family Elapidae, represented by a single species – *Naja haje legioni* [2–4, 12]. Viperidae venoms are particularly rich in enzymes that cause inflammation, coagulation disorders and necrosis. The venom of Elapidae contains neurotoxins and phospholipases responsible for neurological effects that provoke paralysis and respiratory failure [5].

Among the most medically important snake species are *Daboia mauritanica*, *Bitis arietans*, and *Cerastes cerastes. Daboia mauritanica* is particularly abundant in Morocco, and the vast majority of bites reported is attributable to this viper. Chafiq et al. [14] reported four cases of envenomation by *Daboia mauritanica.* Two cases were severe and their clinical symptoms were characterized by thrombocytopenia, low blood pressure, compartment syndrome, and hemorrhagic disorder. In one of these two cases, local necrosis of the thumb, thenar and hypothenar areas, was observed and both cases were submitted to fasciotomy. Envenomation was moderate and minor in the third and fourth cases, respectively. No antivenom was administered in any of these cases [14]. For other viper species – *Vipera latastei*, *Vipera monticola, Echis leucogaster* and *Cerastes vipera* – there are no records of victims and they are probably less involved in bites or not identified.

Despite a significant presence of *Naja haje legionis* in Morocco, bites by this species are likely to be rare [2]. However, we cannot disregard that most neurotoxic envenomations did not reach hospitals, as the mean time between bite and hospital presentation is particularly high. More worrying is the high case fatality rate. It reflected a deficient management of snakebites. The reason may be a strong under-reporting of mild cases, inducing a reduced denominator. It can also result from important delay of hospital presentation that affects the effectiveness of treatment. Finally, the accessibility of antivenom – and its adequacy – should be questioned. The current health policy regarding the management of envenomation recommends providing antivenom only in hospitals of regions with high risk of snakebites. Consequently, antivenom is not available in peripheral health centers. Additionally, FAV-Afrique®, the only antivenom available at the time of the study, does not cover all species of Morocco [15, 16]. Especially two snake species whose venom is particularly toxic and provokes highly severe envenomation: *Daboia mauritanica* and *Ceraste cerastes*. Their bites represent more than 80 % of envenomation. This would explain why the antivenom administration did not appear to reduce significantly the case fatality rate of viper envenomation in Morocco.

The preparation of an appropriate antivenom is based primarily on the selection of venoms used for the immunization of horses to obtain the suitable antibodies against the venom of the species present in the country and, secondly, on the purification of therapeutic immunoglobulins to limit the adverse effects [17–19].

## Conclusion

The specific strategy launched in 2012 had improved the snakebite reporting system. Further efforts are required for a better assessment of needs and an improvement of the management of envenomation in Morocco. Currently, actions should also be focused on the reduction of the delay in hospital admission, sensitizing people to the problem, and accessibility to appropriate antivenom. Training of health personnel on the use of this antivenom must be undertaken as soon as possible.

### Ethics approval

This retrospective study was a summary of snakebites notified to the Centre Anti Poison et de Parmacovigilance du Maroc and did not require ethical clearance.
